# Functional Mobility and Basic Motor Skills in Patients with Multiple Sclerosis and Its Relation to the Anthropometrical Status and Body Composition Parameters

**DOI:** 10.3390/medicina55120773

**Published:** 2019-12-04

**Authors:** Edyta Matusik, Aldona Augustak, Jacek Durmala

**Affiliations:** 1 School of Health Science in Katowice, Department of Rehabilitation, Medical University of Silesia, 40-055 Katowice, Poland; jdurmala@gmail.com; 2 Upper-Silesian Medical Centre, Department of the Therapeutic Rehabilitation, 40-635 Katowice, Poland; aldona.augustak@gmail.com

**Keywords:** multiple sclerosis, physical fitness, body composition, functional mobility, physiotherapy

## Abstract

*Background and objectives*: Patients with multiple sclerosis (MS) have many potential risk factors (spasticity, immobilization, glucocorticoids use) which can deteriorate the anthropometrical status and body composition and may have a potential negative impact on functional mobility and basic motor skill improvement after physiotherapy. The aim of the study was to assess the functional mobility and basic motor skills in patients with MS and to correlate them with disability and anthropometrical status and body composition parameters. *Materials and Methods*: Timed Up-and-Go Test (TUG) and six-min walk test (6MWT) were performed in 36 patients with MS before and after 4 weeks of physiotherapy. Body mass index (BMI), waist-to-height ratio (W/HtR), and waist-to-hip ratio (WHR) were assessed in this group. Body composition was evaluated by bioelectrical impedance analysis (BIA) and fat mass (FAT), fat free mass (FFM), total body water (TBW), and predicted muscle mass (PMM) were expressed as percentage of body mass. Clinical status was assessed by Expanded Disability Status Scale (EDSS) and Ambulatory Index (AI) scales. *Results*: After physiotherapy, there was a significant improvement in functional mobility and basic motor skills assessed by total distance in 6MWT (*p* < 0.001) and in TUG trials (*p* < 0.001). Positive significant correlations were found between the results obtained in both tests (either before and after physiotherapy) vs. FFM, TBW, and PMM, whilst worse results in functional mobility and basic motor skills correlated significantly with higher WHtR, WHR, and FAT (*p* < 0.05). Clinical status (EDSS) was significantly related to the WHtR and body composition parameters with the same manner as the results in the either 6MWT and TUG. However, there were no significant relationships between BMI vs. either clinical status (EDSS, AI) or functional mobility tests results in patients with MS. *Conclusions*: Functional mobility and basic motor skills may be significantly improved during physiotherapy, but they are related to the anthropometrical status and body composition of MS patients. Moreover, disability status is also significantly related to these parameters. Body composition deterioration seems to be the important target for the therapeutic intervention in MS patients. For proper nutritional status assessment in patients with MS, body composition analysis or WHtR instead BMI should to be used.

## 1. Introduction

Multiple sclerosis (MS) is an immune-modulated disease characterized by inflammation and demyelination of the central nervous system [1]. Symptoms of MS include weakness and fatigue, spasticity, abnormal sensation, impaired coordination, mobility and ambulation, and depression. The etiology of MS is not fully understood, but the most popular theory is that MS is provoked by the complex interaction of genetic influences and environmental or modifiable factors which are causative agents in the disease process. One of the recently underlined modifiable factors is obesity [2,3]. A large-scale population-based study conducted in Swedish MS patients showed that subjects whose BMI exceeded 27 kg/m^2^ at age 20 had a 2-fold increased risk of developing MS compared with normal weight subjects [4]. To be able to properly assess nutritional status of MS patients, either standard anthropometry or body composition analysis (by dual energy X-ray absorptiometry (DXA) or bioelectrical impedance analysis (BIA)) have to be performed. However, body composition assessment in people with MS have not been extensively studied. Limited studies have reported that body mass index (BMI) assessment may underestimate adiposity in patients with multiple sclerosis [5]. On the other hand, in the group of MS patients with BMI >25 kg/m^2^, a significantly lower percentage of no evidence of disease activity (NEDA) after beta-interferon therapy than in the normal weight group was noted [6]. Moreover, patients with multiple sclerosis (MS) have many other potential factors (spasticity, immobilization, glucocorticoids use) which can deteriorate the anthropometrical status and body composition and may have the potential impact on the walking ability and other basic motor skills in these persons. Available data showed that only few patients with MS may achieve recommended daily physical activity levels, and their walking ability (measured as step count by 30 min) was most strongly related to gait and balance measures [7]. The most widely use therapeutic method for the improvement of functional mobility in MS patients is physiotherapy. Little is known about the potential relationships between the nutritional status of MS patients and the outcomes of physiotherapy. That is why the aim of this prospective study was to assess the functional mobility and basic motor skills in patients with MS before and after the 4 weeks of rehabilitation and physical activity program. The results obtained were then correlated with both disability and nutritional status assessed by either standard anthropometry and body composition analysis.

## 2. Materials and Methods

### 2.1. Studied Population

**Figure 1:**
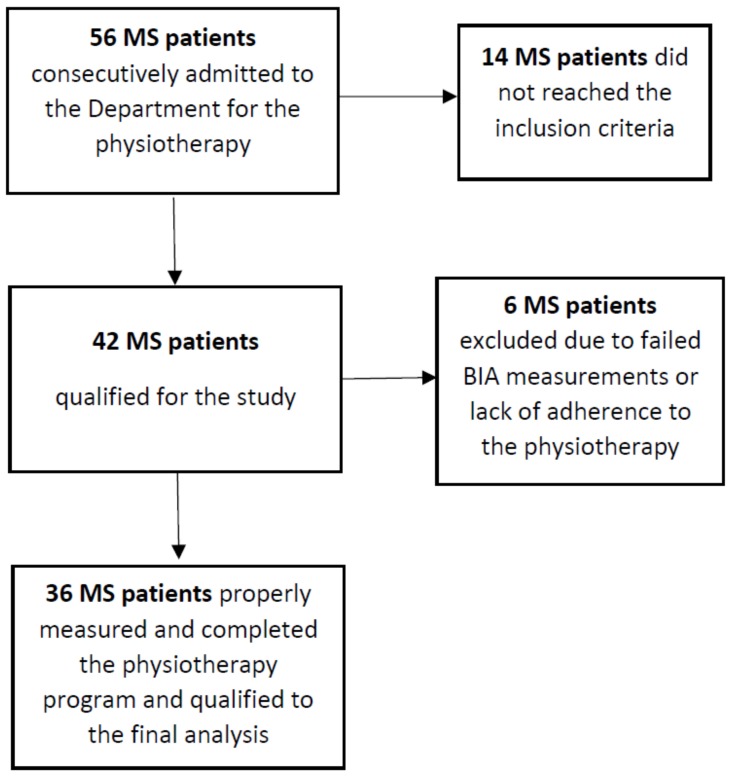
Flow diagram of the recruitment process of the patients. MS, multiple sclerosis; BIA, bioelectrical impedance analysis.

Fifty-six MS patients that were consecutively admitted to the department during 6 months were initially recruited to the study. The details of the patient recruitment process is shown in the flow diagram in Figure 1. The group of subjects finally included to the analysis (*n* = 36, 24 females) had a definite diagnosis of secondary progressive MS according to the McDonald criteria [8] and preservation of at least some ambulatory function (Expanded Disability Status Scale (EDSS) 1.5–6.5, median score 4.5; age 53.83 ± 11.21 years (Table 1)); 9 persons required a walking aid (i.e., a cane or crutches). Subjects had not experienced an exacerbation in the 30 days prior to rehabilitation and testing, and had no other medical conditions that interfered with walking (i.e., cardiological diseases, endocrine disorders, diseases of the musculoskeletal system, orthopedic surgery, current glucocorticoid therapy, or respiratory diseases) were included. A routine medical examination was carried out on each patient on the day of admission. The assessment of neurological status on the EDSS and Ambulatory Index (AI) scale was performed by one neurologist (EM).

### 2.2. Physiotherapy

All subjects included in the study were subjected to rehabilitation lasting 6 days/week for 4 weeks. Every patients started and finished the exercises at the same time. The rehabilitation program conducted in an individual form was dependent on the clinical condition of the patient, but always included exercises: Equivalent, motor coordination, active trunk, and limbs. Each patient underwent training on a treadmill. The intensity of the exercises was individually regulated for each patient and carried out under the supervision of a physiotherapist. Duration, speed, and exercise parameters varied depending on the individual possibilities covered by the study. The total exercise time was 60–90 min. In the afternoon, relaxation exercises, stretching, and exercises lasting 30–45 min were carried out as part of group classes. The physiotherapy content (intensity, types of exercise) was individualized for every patient, depending on the patient’s needs. (i.e., spasticity, gait/balance problem, chronic fatigue, etc.). The duration of individual exercises and the number of rests varied depending on the patient’s exercise tolerance. All patients were followed by the main investigator (E.M.) during the rehabilitation.

### 2.3. Anthropometric Measurements and Body Composition Analysis

A set of anthropometric measurements was recorded at the first day of physiotherapy and performed by the principal investigator (E.M.). Standing height was measured by a wall-mounted Harpender Stadiometer to the nearest 0.1 cm. Weight (in underwear) was measured with an electronic scale with readings accurate to 0.1 kg. Body mass index (BMI) was then calculated, using the standard formula (kilograms per meter squared). Waist and hip circumferences were also measured, and both waist-to-hip (WHR) and waist-to-height ratio (W/HtR) were calculated. Body composition parameters: Fat mass (FAT), fat-free mass (FFM), predicted muscle mass (PMM), and total body water (TBW) were assessed (in kilograms [kg] or as percentage of body weight [%]) based on bioelectrical impedance using a segmental body composition analyzer (BC-420MA Tanita Europe BV, Hoofddorp, The Netherlands).

### 2.4. Functional Mobility and Basic Motor Skills Testing

To assess the functional mobility and basic motor skills the 6-min walk test (6MWT) and the Timed Up-and-Go Test (TUG) were administered by the same investigator (A.A.). 6MWT was made according the methodology described by Goldman et al. [9]. Subjects walked, at maximal effort, back and forth in a 30-m hallway turning round cones, and were allowed to use their habitual assistive devices. Total walked distance and heart rate (HR) at baseline and at the end of the attempt were registered. In the TUG, the timed performance of getting up from a chair, walking 3 m, turning around, and walking back to sit down again was assessed [10]. Three trials of the TUG test were performed. The time was recorded in seconds, with the fastest of the three trials used for analysis. Both of the tests were carried out twice before and after the rehabilitation program and were performed under the same conditions.

### 2.5. Ethical Considerations

The study was approved by the Ethics Committee of the Medical University of Silesia (Approval No. KNW/0022/KB/179/17 from the 30^th^ of June 2017). All participants gave informed consent. Patient rights were also approved according to the Helsinki Declaration.

### 2.6. Statistical Analysis

All data were distributed normally (assessed by Kolmogorov–Smirnov test). Differences in continuous variables between before vs. after physiotherapy were assessed by paired Student’s *t*-test for independent variables with non-equal variances. One-way analysis of variance (ANOVA) was used to analyze any significant difference among the four nutritional status subgroups, i.e., underweight, normal weight, overweight, and obesity. Correlations between continuous parametrical were based on linear Pearson’s correlation coefficient. All statistical analysis was made with the Statistica™ 12 PL software and a *p*-value less than 0.05 was considered statistically significant.

## 3. Results

Baseline characteristics and anthropometric measurements of all studied MS patients are reported in Table 1.

Functional mobility and basic motor skills in patients with MS, assessed by total distance in 6MWT and timed performance in TUG, improved significantly after 4 weeks of physiotherapy (Table 2). The mean difference of total walked distance in 6MWT after physiotherapy was 14.05 ± 22.43 m, while mean TUG difference was recorded as 0.58 ± 1.11 s.

For the next stage of the analysis, the study group was further divided according to the nutritional status, i.e., underweight (BMI < 18.5 kg/m^2^, *N* = 2), normal weight (BMI = 18.5−24.9 kg/m^2^, *N* = 17), overweight (BMI = 25−29.9 kg/m^2^, *N* = 10), obesity (BMI ≥ 30 kg/m^2^, *N* = 7).

**Figure 2:**
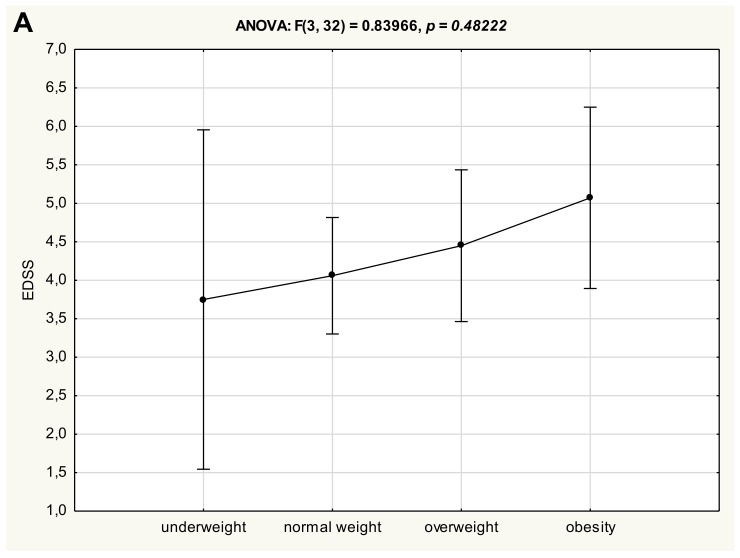

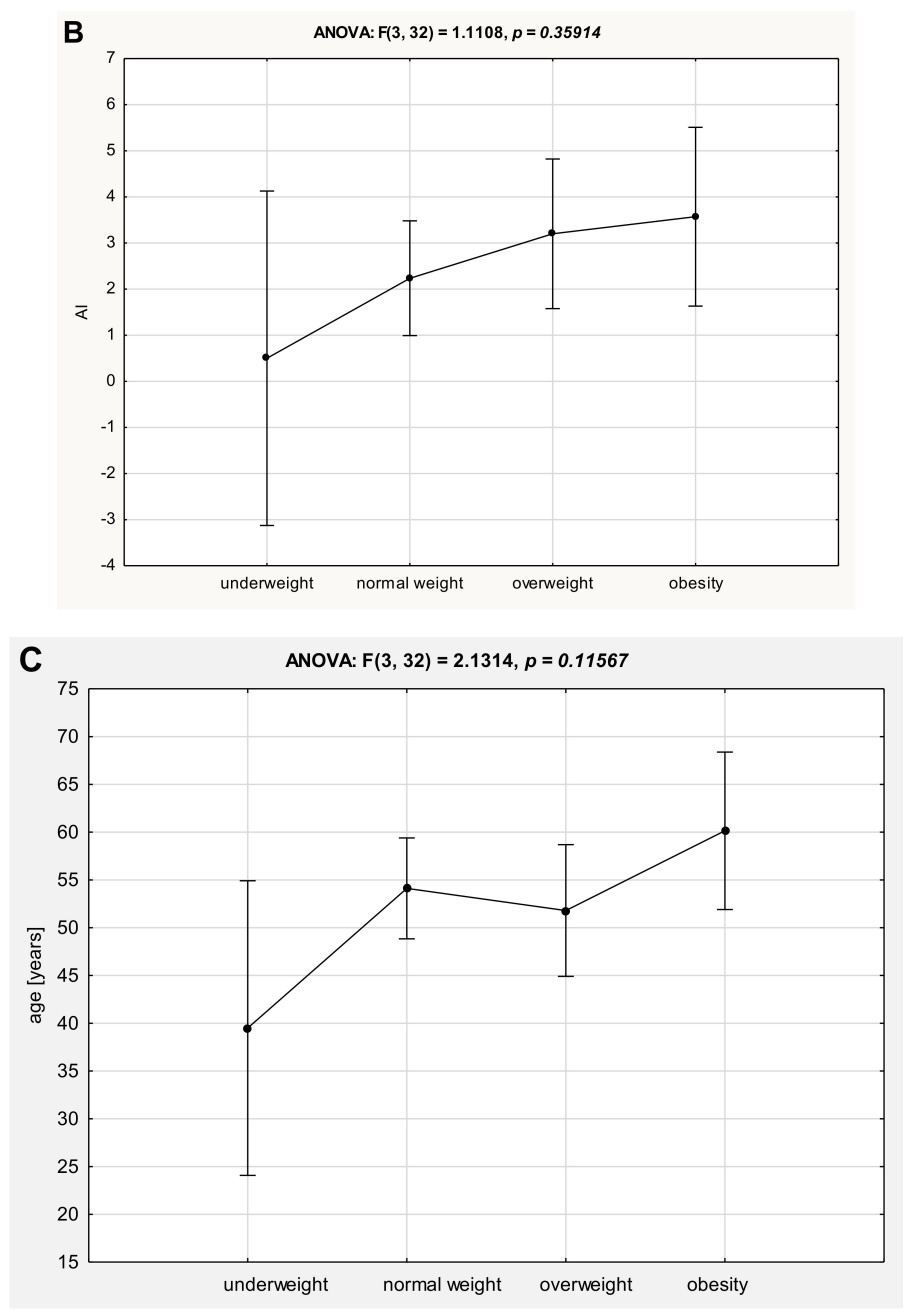

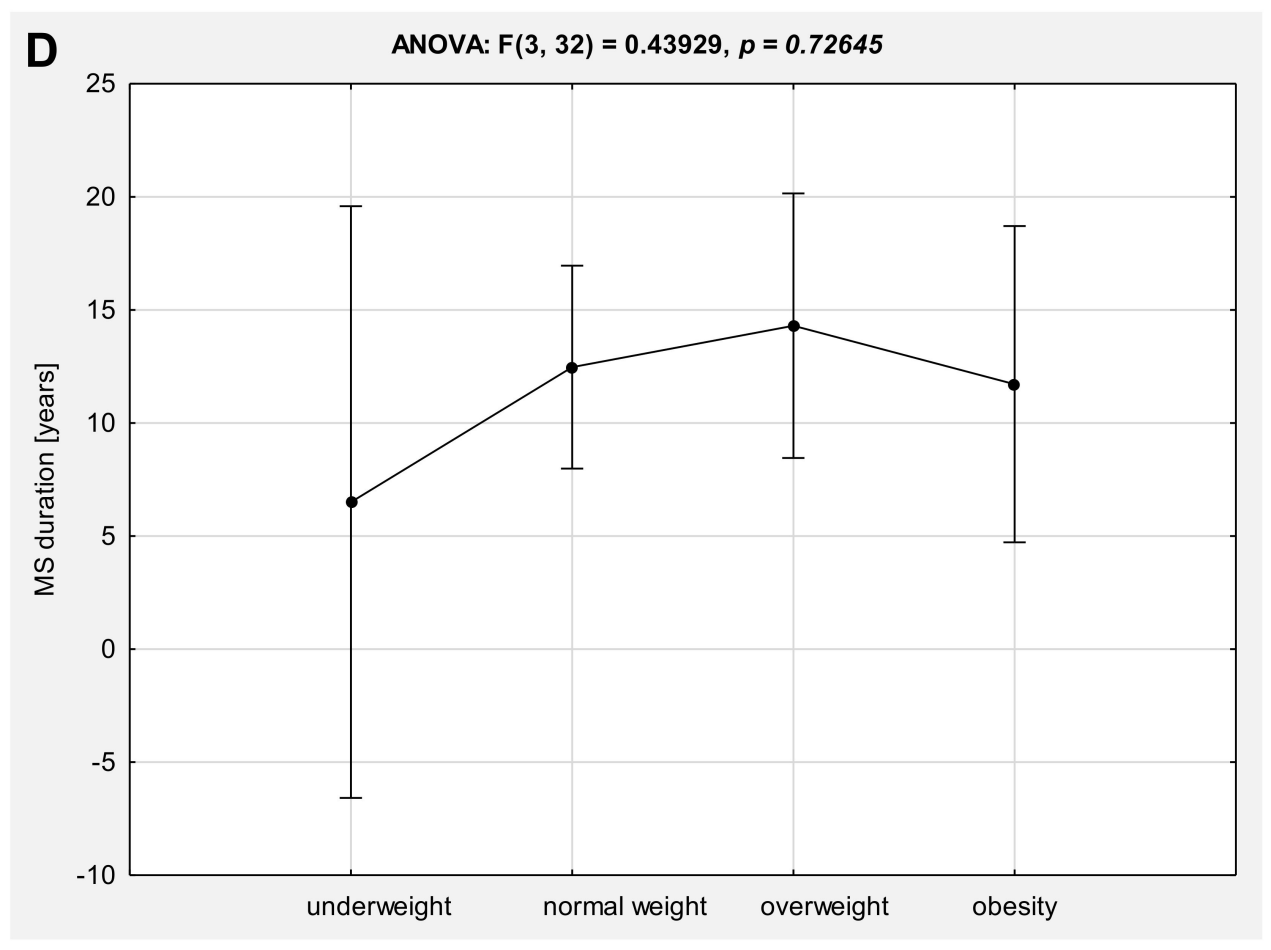
Clinical status of MS patients expressed as EDSS (**A**) and AI (**B**), age (**C**) and MS duration (**D**) after the stratification to the different nutritional status subgroups.

One-way analysis of variance (ANOVA) did not reveal significant differences in clinical status of patients with multiple sclerosis between four anthropometrical subgroups, neither for EDSS (Figure 2A) nor AI (Figure 2B). However, a trend of gradual deterioration in the clinical status (assessed either as EDSS or AI) of patients with MS was observed, along with an increase in BMI value, but these differences did not reach statistical significance. Otherwise, there were no significant differences in either age (Figure 2C) or duration of the disease (Figure 2D) in reference to the anthropometrical status.

However, further analysis revealed significant baseline correlations between EDSS vs. WHtR and body composition parameters FAT, FFM, and PMM, while there were no significant correlations between BMI, waist and hip circumferences, WHR vs. EDSS or AI (Table 3).

Table 4 summarizes the relationship between the age and clinical status of MS patient with the results obtained in 6MWT and TUG trials before and after physiotherapy. The age of MS patients correlates in a significant expected manner (negatively) with 6MWT total distance either before and after physiotherapy, as well as with the TUG trials (positively). The same significant relationships were found in the cases of EDSS and AI. Moreover, the clinical status expressed as either EDSS or AI was significantly related to the heart rate at baseline and at the end of every 6MWT trial.

Table 5 is a summary of the relationship between the anthropometrical standard measurements (BMI, waist and hip circumferences, WHtR, and WHR) with the results obtained in the 6MWT and TUG trials before and after physiotherapy. BMI and hip circumference did not correlate significantly with parameters from the functional mobility tests performed. However, there was a significant positive correlation between waist circumference and HR at the end of both before and after physiotherapy performed in the 6MWT test. There were also significant correlations between abdominal obesity diagnosed as WHtR and 6MWT distance (negative), as well as TUG trials (positive).

Body composition parameters (FAT, FFM, TBW, and PMM) findings were similar to the WHtR findings and reached the same significance. Larger adiposity has a significant negative impact on the mobility functioning and basic motor skills in the studied MS population (Table 6).

Moreover, to verify if the rehabilitation-induced improvement in 6MWT and TUG was influenced by the anthropometrical indices, correlations between changes in the 6MWT and TUG results and the indices were calculated. However, no statistically significant relationships were found.

## 4. Discussion

The results of this prospective study confirm the significant correlations between clinical disability (expressed as EDSS) vs. nutritional status (expressed as WHtR and FAT, FFM, and PMM), while there were no significant correlations between routinely performed BMI, waist and hip circumferences, and WHR. Our findings confirm the data from the study by Pilutti et al., which showed that BMI assessment may cause underestimation of adiposity in patients with MS comparing with body composition assessed by DXA [4]. Moreover, in our study one-way analysis of variance (ANOVA) did not reveal significant differences in clinical status of patients with multiple sclerosis between four anthropometrical subgroups (stratified by BMI). There was only a trend of gradual deterioration in the clinical status (assessed either as EDSS or AI) of patients with MS that was observed, along with an increase in BMI value, but these differences did not reach statistical significance.

Areal BMD (aBMD) measured by dual-energy X-ray absorptiometry (DXA) is currently the gold standard, not only for the diagnosis of osteoporosis, but also for body composition evaluation. However, a noninvasive body composition assessment technique is currently available based on bioelectrical impedance analysis (BIA). A good correlation between BIA and DXA has been reported in estimating adiposity in the different groups of patients [11,12]. BIA is relatively simple, quick, non-invasive, and readily accessible compared to other more sophisticated methods, such as quantitative computed tomography (qCT) or DXA. In the present study, body composition parameters FAT, FFM, TBW, and PPM were significantly related to the total walked distance assessed in the 6MWT and with the level of functional mobility based on the timed performance of the TUG trials. These relationships persisted also after 4 weeks of the rehabilitation program. Body composition analysis by BIA in MS patients has only been used in the study conducted by Bromley et al. [13] in only 20 MS patients. Their work focused mainly on nutritional intake rather than nutritional status, and they found a significant correlation between the diet comprising saturated fat (positive) and carbohydrates (negative) with distance covered during the 6MWT. However, body fat percentage assessed by BIA was only shown as a mean result for the studied population, but the authors did not show any detailed results related to BIA. Another aspect of body composition in MS patients is the possible higher risk for sarcopenia related to the level of disability. Our study showed significant positive correlations of all lean mass-related parameters (FFM, TBW, and PMM) to the longer total walking distance in 6MWT and shorter TUG trials. It is also important to realize that lean mass is strongly related to the bone mineral density (BMD), either in whole body and lumbar spine projection [14]. To summarize, our study strongly suggests that larger adiposity has a significant negative impact on the mobility functioning and basic motor skills in the studied MS population, and may influence the physiotherapy outcomes in this patient group. However, in our study MS patients, there were no significant relations between the level of functional mobility and basic motor skill improvements (expressed as delta of total distance in 6MWT and delta TUG), and the baseline disability level (EDSS, AI) and the baseline nutritional status. On the other hand, our data proved the efficacy of the 4-week rehabilitation program on the functional capacity in the MS patients. A recent study by Feys et al. showed that exercise therapy in persons with MS not only improved functional mobility and aerobic capacity, but also had a positive impact on visuospatial memory, fatigue, and quality of life [15].

Based on our study, the only anthropometrical parameter that seems to be useful in nutritional status assessment in MS patients is the waist-to-height ratio (WHtR). WHtR correlated significantly with both total walked distance in 6MWT and TUG trials time, with the same manner as percentage of body fat mass (FAT) assessed by BIA. WHtR is now widely studied, with the aim to find relatively simple parameters of fat tissue distribution in connection with visceral obesity and its comorbidities. A recent analysis showed WHtR as a better parameter for the prognosis of visceral obesity and its comorbidities as the waist-to-hip ratio (WHR) [16]. In our study, WHR was only significantly related to heart rate (HR) at the end of the 6MWT attempt independently from physiotherapy. The above findings confirm great importance of body fat distribution, with the special risk for abdominal/visceral obesity, which cannot be properly assessed by BMI itself.

Limitations of this study must be acknowledged. First, the use of a cross-sectional design with a relatively small number of participants (especially with underweight) can provide only correlations and not direct causative findings. Second, participants with better clinical status (lower EDSS scores) may have more social (dietary and behavioral) opportunities to change their body composition, by interaction with friends and family and at work, which allows for more frequent episodes of dining outside of the house, and have still quite normal daily physical activity. In contrast, participants with higher EDSS scores may be dependent on a caretaker for meal preparation and definitely have lower physical activity, and are probably have higher risk for body composition deterioration (adiposity or sarcopenia). The third limitation is the lack of a control group; however it is difficult to avoid this (from the ethical point of view) based on the routine hospitalization. That is why future studies will have to focus on the specific MS severity subgroups of the patients, taking into account other potentially confounding factors.

## 5. Conclusions

Functional mobility and basic motor skills may be significantly improved during physiotherapy, but they are related to the anthropometrical status and body composition of MS patients. Moreover, disability status is also significantly related to these parameters. Body composition deterioration seems to be the important target for therapeutic intervention in this group of patients. For proper nutritional status assessment in patients with MS, body composition analysis or WHtR instead of BMI should to be used.

## Figures and Tables

**Table 1:** Clinical and anthropometrical characteristics of the study population.

	Studied Population *N* = 36 (F/M 24/12)			
	Mean	Minimum	Maximum	SD
**EDSS**	4.4	1.5	6.5	1.5
**AI**	2.7	0	9	2.5
**Height (cm)**	167.4	152.1	190.1	9.5
**Weight (kg)**	72.4	44.6	94.1	12.5
**BMI (kg/m^2^)**	25.89	17.2	39.2	4.6
**Waist c. (cm)**	93.8	70.2	117.3	11.8
**Hip c. (cm)**	102.4	85.1	127.3	8.3
**WHtR**	0.56	0.42	0.69	0.08
**WHR**	0.92	0.73	1.03	0.08
**FAT (kg)**	22.7	6.3	41.4	8.5
**FAT (%)**	30.9	12.9	45.2	8.9
**FFM (kg)**	49.9	36.5	70.5	9.7
**FFM (%)**	69.2	54.8	87.1	8.9
**TBW (kg)**	35.7	26	51.5	6.7
**TBW (%)**	49.4	38.8	60.6	5.9
**PMM (kg)**	47.5	34.7	67.3	9.3
**PMM (%)**	65.8	51.9	82.7	8.6

Abbreviations: EDSS—Expanded Disability Status Scale; AI—Ambulatory Index; BMI—body mass index; WHtR —waist-to-height ratio; WHR—waist-to-hip ratio; FAT—fat mass; FFM—fat-free mass; TBW—total body water; PMM—predicted muscle mass.

**Table 2:** Total walking distance [m] of the 6-min walk test (6MWT) and Timed Up-and-Go Test (TUG) result [s] at baseline vs. after physiotherapy.

	At Baseline (Mean ± SD)	After Physiotherapy (Mean ± SD)	Significance
**6MWT (m)**	289.38 ± 137.67	303.43 ± 141.14	*p* < 0.001
**TUG (s)**	10.09 ± 5.24	9.47 ± 4.52	*p* < 0.01

**Table 3:** Correlations of clinical status with individual anthropometric and body composition parameters in studied MS population (* *p* < 0.05).

	BMI	Waist c.	Hip c.	WHtR	WHR	FAT (%)	FFM (%)	PPM (%)
**EDSS**	0.314	0.245	0.250	0.423 *	0.114	0.408 *	−0.407 *	−0.398 *
**AI**	0.328	0.165	0.296	0.330	−0.040	0.308	−0.307	−0.301

Abbreviations: EDSS—Expanded Disability Status Scale; AI—Ambulatory Index; BMI—body mass index; WHtR —waist to height ratio; WHR—waist to hip ratio; FAT—fat mass; FFM—fat-free mass; TBW—total body water; PMM—predicted muscle mass.

**Table 4:** Correlations of age and clinical status of MS patients with results obtained in individual 6MWT and TUG trials before (1) and after (2) physiotherapy (* *p* < 0.05; ** *p* < 0.001; ^#^ *p* < 0.0001; ^##^ *p* < 0.00001).

	6MWT 1 HR at Baseline	6MWT 1 HR at the End	6MWT 2 HR at Baseline	6MWT 2 HR at the End	6MWT 1 Distance	6MWT 2 Distance	TUG 1	TUG 2
**age**	0.169	0.139	0.114	0.010	−0.561 **	−0.558 **	0.380 *	0.389 *
**EDSS**	0.357 *	0.489 *	0.254	0.264	−0.911 ^##^	−0.890 ^##^	0.645 ^#^	0.644 ^#^
**AI**	0.273	0.532 **	0.153	0.216	−0.816 ^##^	−0.783 ^##^	0.555 **	0.562 **

Abbreviations: EDSS—Expanded Disability Status Scale; AI—Ambulatory Index; 6MTW—6-min walk test; HR—heart rate; TUG—Timed Up-and-Go Test.

**Table 5:** Correlations of individual anthropometric parameters with the results obtained in individual 6MWT and TUG trials before (1) and after (2) physiotherapy (* *p* < 0.05).

	6MWT 1 HR at Baseline	6MWT 1 HR at the End	6MWT 2 HR at Baseline	6MWT 2 HR at the End	6MWT 1 Distance	6MWT 2 Distance	TUG 1	TUG 2
**BMI**	0.164	0.162	0.129	0.139	0.148	0.115	0.162	0.156
**Waist c.**	0.323	0.358 *	0.294	0.375 *	0.210	0.182	0.289	0.272
**Hip c.**	0.118	0.089	0.169	0.198	0.219	0.197	0.251	0.246
**WHtR**	0.228	0.251	0.199	0.224	−0.348 *	−0.322 *	0.351 *	0.342 *
**WHR**	0.323	0.410 *	0.239	0.329 *	0.095	0.076	0.182	0.164

Abbreviations: 6MWT—6-min walk test; HR—heart rate; TUG—Timed Up-and-Go Test; BMI—body mass index; WHtR —waist-to-height ratio; WHR—waist-to-hip ratio.

**Table 6:** Correlations of body composition parameters with the results obtained in individual 6MWT and TUG trials before (1) and after (2) physiotherapy (* *p* < 0.05).

	6MWT 1 HR at Baseline	6MWT 1 HR at the End	6MWT 2 HR at Baseline	6MWT 2 HR at the End	6MWT 1 Distance	6MWT 2 Distance	TUG 1	TUG 2
**FAT [%]**	0.145	0.061	0.186	0.136	0.417 *	0.415 *	0.383 *	0.372 *
**FFM [%]**	−0.145	0.060	−0.186	0.136	0.412 *	0.414 *	−0.382 *	−0.372 *
**TBW [%]**	−0.145	0.009	−0.179	0.102	0.380 *	0.377 *	−0.352 *	−0.341 *
**PMM [%]**	−0.141	0.053	−0.182	0.133	0.414 *	0.412 *	−0.379 *	−0.369 *

Abbreviations: 6MWT—6-min walk test; HR—heart rate; TUG—Timed Up-and-Go Test; FAT—fat mass; FFM—fat-free mass; TBW—total body water; PMM—predicted muscle mass.
